# FOXP3+ regulatory T cells and the immune escape in solid tumours

**DOI:** 10.3389/fimmu.2022.982986

**Published:** 2022-10-13

**Authors:** Yiran Qiu, Shouyu Ke, Jieqiong Chen, Zhizhen Qin, Wenle Zhang, Yaqin Yuan, Dehua Meng, Gang Zhao, Kejin Wu, Bin Li, Dan Li

**Affiliations:** ^1^ Department of Breast Surgery, Obstetrics and Gynecology Hospital, Fudan University School of Medicine, Shanghai, China; ^2^ Center for Immune-Related Diseases at Shanghai Institute of Immunology, Department of Respiratory and Critical Care Medicine of Ruijin Hospital, Shanghai Jiao Tong University School of Medicine, Shanghai, China; ^3^ Department of Gastrointestinal Surgery, Renji Hospital, Shanghai Jiao Tong University School of Medicine, Shanghai, China; ^4^ Department of Orthopedics, Zhongshan Hospital, Fudan University School of Medicine, Shanghai, China; ^5^ Institute of Arthritis Research, Guanghua Integrative Medicine Hospital, Shanghai University of Traditional Chinese Medicine, Shanghai, China; ^6^ Department of Integrated TCM & Western Medicine at Shanghai Skin Disease Hospital, School of Medicine, Tongji University, Shanghai, China; ^7^ Department of General Surgery, Huashan Hospital, Fudan University, Shanghai, China

**Keywords:** regulatory T Cells, FOXP3+, tumour microenvironment, immune escape, immune metabolism

## Abstract

FOXP3+ regulatory T (Treg) cells play critical roles in establishing the immunosuppressive tumour microenvironment, which is achieved and dynamically maintained with the contribution of various stromal and immune cell subsets. However, the dynamics of non-lymphoid FOXP3+ Treg cells and the mutual regulation of Treg cells and other cell types in solid tumour microenvironment remains largely unclear. In this review, we summarize the latest findings on the dynamic connections and reciprocal regulations of non-lymphoid Treg cell subsets in accordance with well-established and new emerging hallmarks of cancer, especially on the immune escape of tumour cells in solid tumours. Our comprehension of the interplay between FOXP3+ Treg cells and key hallmarks of cancer may provide new insights into the development of next-generation engineered T cell-based immune treatments for solid tumours.

## Introduction

Tumour is a leading cause of death and a significant barrier to the increasing life expectancy worldwide (1, 2). It remains largely an incurable disease, urging us to explore the mystery of the tumour tissue microenvironment. Although the comprehensive mechanisms for tumour progression are still unclear, we have known for more than one decade that the insufficient anti-tumour immunity is caused by regulatory T (Treg) cell-mediated immunosuppression (3). Hanahan and Weinberg have previously published landmark reviews on The Hallmarks of Cancer to gather and categorize the knowledge of cancer into several hallmarks, leading to a systematic understanding of cancer occurrence and development as well as guiding the research direction in past decades (4, 5). In 2022, Hanahan has added four proposed emerging hallmarks and enabling characteristics, “unlocking phenotypic plasticity,” “nonmutational epigenetic reprogramming,” “polymorphic microbiomes,” and “senescent cells” in addition to the ten well-established ones, including “sustaining proliferative signaling,” “deregulating cellular metabolism,” “resisting cell death,” “genome instability and mutations,” “inducing or accessing vasculature,” “activating invasion and metastasis,” “tumour-promoting inflammation,” “enabling replicative immortality,” “avoiding immune destruction” and “evading growth suppressors” (6). In this review, we are going to discuss the potential connection between non-lymphoid FOXP3+ regulatory T cell dynamics and the new emerging and well-established hallmarks of cancer, especially on the immune escape of solid tumours.

Treg cells, also known as suppressor T cells, are a subpopulation of T cells that modulate the immune system (7). The lineage determining transcription factor, FOXP3 forms a large molecular complex with multiple transcription factors and enzymatic subunits to dynamically regulate the development and function of regulatory T cells (8–14). FOXP3+ Treg cells play essential roles in maintaining immune homeostasis in healthy people (15). However, tumour-infiltrating Treg cells have strong immunosuppressive function, which may promote the immune escape of cancer cells and the occurrence and development of tumours (16, 17). Meanwhile, the tumour-derived factors may also mutually modulate the induction, migration, and immunosuppressive function of FOXP3+ Treg cells (17).

## Mechanisms of FOXP3+ Treg cell-mediated immune homeostasis and anti-tumour immunity in solid tumour microenvironment

Tumour progression is not only related to the anabolic metabolism of tumour cells themselves, but also to the extracellular matrix in the tumour microenvironment (TME). Within TME, stromal cells maintain tissue homeostasis which favours the growth of tumours, while Treg cells dominate the formation of immunosuppressive TME, resulting in the failure of launching effective anti-tumour responses (18). Although the ablation of Treg cells can eradicate tumours rapidly, severe autoimmune and inflammatory complications are developed due to the loss of Treg cell function (19). During the development of tumours, Treg cells proliferate and undergo functional maturation, which are promoted by metabolites produced by tumour cells. Therefore, a deep understanding of underlying mechanisms of FOXP3+ Treg cells mediated immune homeostasis and anti-tumour immunity is required for developing more effective anti-tumour immunotherapies.

The main function of Treg cells is to maintain the immune balance and promote tissue homeostasis. In the tumour microenvironment, Treg cells have multiple functions and could suppress the anti-tumour response through several mechanisms. Treg cells express immune inhibitory receptors and ligands such as CTLA-4, PD-1, and PD-L1 (20). In addition, Treg cells can express high-affinity IL-2 receptor subunit CD25, which may deplete the pro-inflammatory factor IL-2 in TME (21). Treg cells also express cell surface ectonucleotidases CD39 and CD73, which degrade extracellular ATP into adenosine, leading to the functional immunosuppression of target cells (22). FOXP3+ Treg cells may also secrete anti-inflammatory factors (TGF-β, IL-10, and IL-35), perforins, and granzymes to inhibit or kill T cells, NK cells, and antigen-presenting cells (23). Blimp1 in Treg cells affects the growth rate of tumours dependent on the expression of Eomesodermin (Eomes), and causes changes in CD45 cells’ type I interferon in TME, resulting in the changes of the downstream angiogenic related genes, MHC I and MHC II molecules, and antigens, thereby altering the activity of tumour immune cells and immunogenicity of the tumour (24).

## An updated view of FOXP3+ Treg cells and solid tumour microenvironment

**Figure 1:**
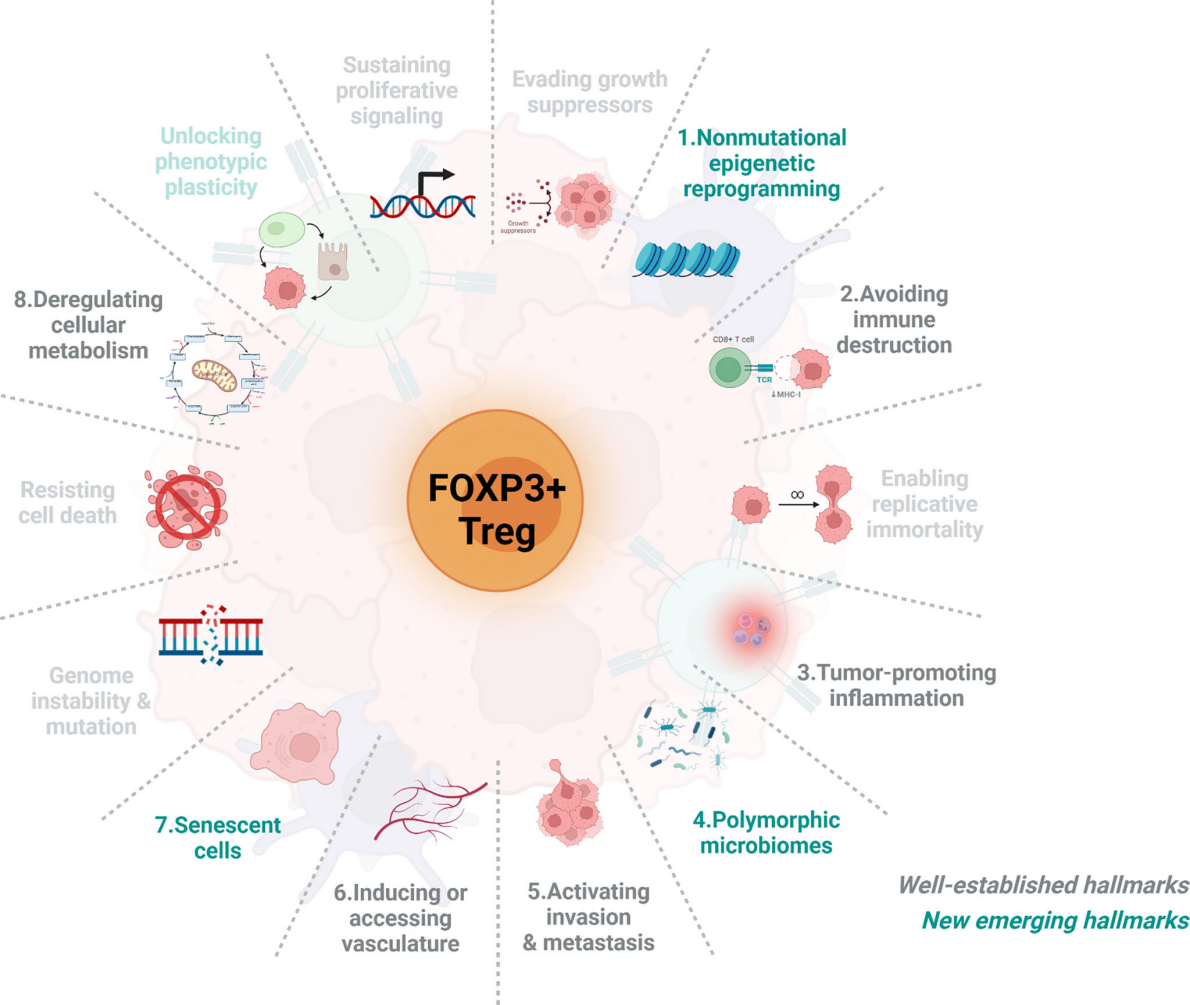
The schematic representation of the associations between FOXP3+ Treg cells and eight cancer hallmarks. Over the past decades, our understanding of cancer has evolved tremendously. Recently, Hanahan and Weinberg have categorized and summarized knowledge of cancers into 14 hallmarks, including 10 well-estlabished hallmarks (grey) and 4 new emerging hallmarks (green). Here we briefly introduce the connection between Treg cells dynamics and the feature of eight either well-established or new emerging cancer hallmarks including 1) nonmutational epigenetic reprogramming, 2) Avoiding immune destruction, 3) tumour-promoting inflammation, 4) polymorphic microbiomes, 5) activating invasion & metastasis, 6) inducing or accessing vasculature, 7) senescent cells, and 8) deregulating cellular metabolism. As very few papers have reported the association of Treg cells with the remaining hallmarks and thus will not be included.

This review will focus on the functional regulation of tumour-infiltrating FOXP3+ Treg cell dynamics in accordance with well-established and new emerging cancer hallmarks in order to provide a more comprehensive understanding of the mutual regulation between FOXP3+ Treg cell dynamics and solid tumour progression (as shown in **Figure 1**).

### FOXP3+ Treg cells and the new hallmark: Nonmutational epigenetic reprogramming

Douglas Hanahan has proposed “nonmutational epigenetic reprogramming” as one of the emerging hallmarks of cancer (6). It has been reported that epigenetic changes within TME, such as excessive alteration of DNA methylation, histone modification, chromatin accessibility, and posttranslational modification, significantly contribute to the development and progression of malignant tumours (6).

Malignant cells apply epigenetic modifications to dysregulate the expression of certain ligands and affect the immunosuppressive ability of Treg cells. One persuasive example is in lymphoma. Pharmacologic inhibition/blockade of Histone deacetylase (HDAC) 11 enhances the expression of OX40L in Hodgkin lymphoma (HL) cells, and the HDAC inhibitor-induced OX40L inhibits the immunosuppressive function of interleukin 10 (IL-10)-producing Treg cells and alters cytokine secretion of HL cells to favour a Th1- and Th17-type response (25). Moreover, studies have reported that OX40 triggers the inhibition of FOXP3 gene expression and the TGF-β–induced conversion of CD4+ naive T cells into CD4+ CD25+ FOXP3+ Treg cells (26, 27).

Cancer epigenetic reprogram also modulates Treg cell functions *via* PD-L1 expression. The interaction between PD-1 and PD-L1 negatively impacts the functions of effector and immunosuppressive T cells. Thus, blocking PD-1/PD-L1 may reactivate anti-tumour T cell immunity, thereby inhibiting tumour growth. Both HDAC inhibitors and DNA-methyltransferase-targeted inhibitors may increase PD-L1 expression in various tumours (28–31). Combination of epigenetic modulators with anti-PD-1/PD-L1 antibodies emerges as promising therapeutics for cancer treatment (29, 32–34). Our recent study has found that gallic acid, a small molecule compound found in traditional Chinese medicine, when combined with anti-PD-1 antibody, significantly dampen tumour- infiltrating FOXP3+ Treg cell function by impairing PD-1/PD-L1 signaling and Foxp3 stability in colorectal cancer (CRC) model (35). By inhibiting the inducible expression of PD-L1, the metabolic molecule L-5-hydroxytryptophan could also stimulate anti-tumour immunity (36). In Treg cells, the PD-1/PD-L1 axis inhibits the phosphorylation of ZAP70 and AKT through phosphorylation of SHP2, which are well established in CD8+ T cells (37–39). Interaction between malignant cells and Tregs is mediated in part through PDL1 and PD1 and epigenetic mechanisms modulated PD-L1 expression level (31, 40). The increase of PD-L1 by malignant cells enhances PD-L1 and PD-1 interactions, which might inhibit both effector T cells and Treg cells, suggesting the epigenetic inhibition might affect anti-tumour immune response. Therefore, the balance of PD-1 expressed by effector T cells and Treg cells in TME, might be considered in the combination of PD-1/PD-L1 blockage and epigenetic inhibition (41).

Enhancer of zeste homologue 2 (EZH2) is a histone H3K27 methyltransferase of the polycomb repressor complex 2 (PRC2) (42). Blockade of this epigenetic regulator dramatically represses tumour *via* a T cell-dependent mechanism. EZH2 inhibition, either pharmacologically or genetically, destabilizes FOXP3 expression in Treg cells and specifically reprograms tumour- infiltrating Treg cells through driving the expression pro-inflammatory genes (e.g., IL-2) while inhibiting key immunosuppressive genes such as IL-10 and TGF-β (43). Treg cell reprogramming toward pro-inflammatory activities is critical for the efficacy of anti-tumour immune responses and enhancing immunotherapy.

### FOXP3+ Treg cells and the immune escape of cancer

The induction and recruitment of immunosuppressive Treg cells is one of the critical processes involved in the acquisition of immune escape in cancer. First, cancer cells can establish immunosuppressive microenvironment by recruiting Treg cells into the tumour through multiple mechanisms. Specific combination of chemotactic molecules and their receptors are engaged in this process. CCR8, exhibiting chemotaxis to CCL1 (44), is a selectively upregulated molecule in intratumoural Treg cells (45, 46). In mouse and human tumour tissues, CCR8+ Treg cells account for 30% -80% of total tumour-infiltrating Treg cells, while that accounts for less than 10% in other tissues (47). Increased Helios+ CCR8+ Treg cell frequency in pancreatic ductal adenocarcinoma (PDAC) is associated with an invasive phenotype and poor survival (48). Anti-CCR8 monoclonal antibodies and anti-CCR8 antibody with Fc-dependent ADCC (antibody dependent cellular cytotoxicity) selectively depletes tumour-infiltrating Treg cells due to significantly increased CCR8 expression by the activated Treg cells in TME, resulting in a durable anti-tumour immune response without deleterious autoimmunity and the anti-tumour effects can be synergized with PD-1 blockers (47, 49, 50). CCR4, binds to CCL22 and CCL17, is another crucial chemokine receptor mediating Treg cells trafficking into the TME (51, 52). Increased CCR4 expression is observed in activated Treg cells. Inhibition of CCR4 has been shown to reduce Treg cells accumulation, potentiate anti-tumour immune activity, sensitize tumours to PD-1 blockade and improve survival (53–56). CCL5, activated by cancer FOXP3, is responsible for FOXP3 + Treg cells infiltration in pancreatic ductal adenocarcinoma (57). Moreover, CCR5-dependent Treg cell recruitment is reportedly in colon cancer and melanoma (58, 59). Beyond the traditional chemotactic mediators, recent studies have also demonstrated that the G protein-coupled receptor 15 (GPR15), an unconventional chemokine receptor, directs the infiltration of Treg cells into the colon and subsequently promotes immune evasion of colorectal cancer (60).

Second, Treg cells may also accumulate in tumour to mediate immunosuppression by conversion of conventional CD4 T (Tconv) cells to Treg cells. Specific cytokines and growth factors in TME are capable to initiate this process. Indoleamine 2,3-dioxygenase (IDO) expressed by cancer cells directly amplifies Treg cells by transforming CD4+CD25-T cells to CD4+CD25+ Treg cells (61). Tumour-derived TGF-β, IL-10, and vascular endothelial growth factor (VEGF) promote the expansion of natural Treg (nTreg) cells assisted by antigen-presenting cells (APCs) in a tolerogenic manner (62). Tumour-infiltrating Treg cells directly promote tumour immune evasion in multiple ways. One of the most important mechanisms is the expression of checkpoint suppressor molecules such as CTLA-4, PD-1, TIM-3, LAG-3, and TIGIT (17, 63). Treg cells function to bind and block corresponding ligands on APCs through these co-inhibitory receptor molecules, thereby inhibiting the maturation and function of APCs. CTLA-4 is constitutively expressed on Treg cells. Compared to CD28, CTLA4 has a higher affinity for CD80/CD86 (64). Once bound, Treg cells can reduce APCs’ expression of CD80/CD86 *via* CTLA-4–dependent trogocytosis (65–67). This CD80/CD86 reduction on APCs also upregulates free PD-L1 on APCs (67). Treatment with CTLA-4 blockers significantly enhances anti-tumour immunity (68). LAG3 expressed by Treg cells can inhibit the expression of MHC II in dendritic cells (DCs) (69). However, it has been demonstrated that the primary fuction of MHC II in LAG-3 immunosuppression is actually mediated by the fibrinogen-like protein 1 FGL1 (70).

Additionally, Treg cells express high levels of CD39 and CD73. These two ecto-nucleotidases contribute to the conversion of ATP released from apoptotic Treg cells into adenosine (71). This directly inhibits the growth of effector T cells and the function of dendritic cells through the adenosine A2A receptor (A2AR) (71). CD39 and CD73 expression in Treg cell are increased in human cancers (72). Blockade of adenosine A2A receptor has been shown to significantly reduce Treg cells and boosts the anti-tumour activity (73). Targeting CD39 by antisense oligonucleotide also represents a promising strategy (74).

**Figure 2:**
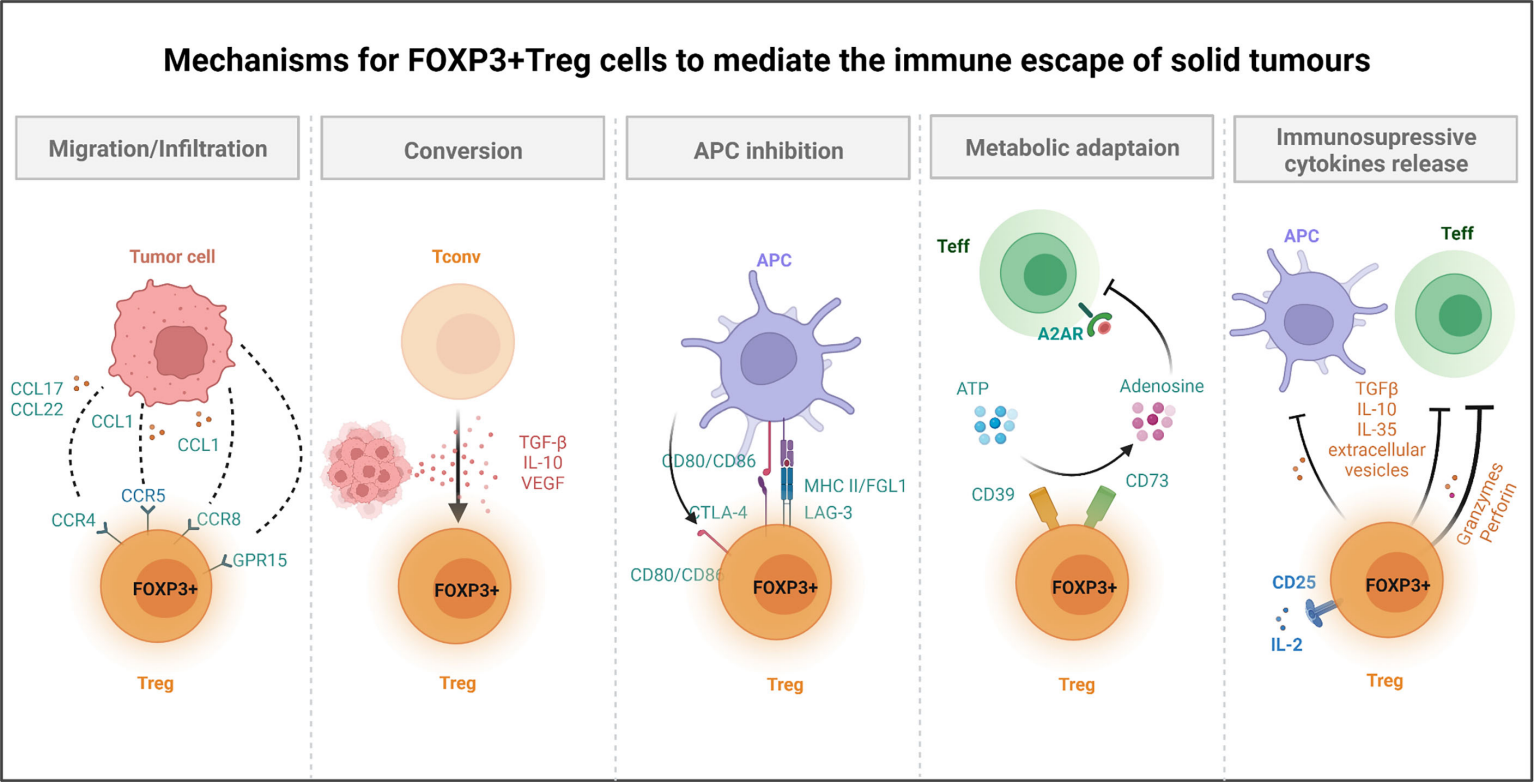
Mechanisms for FOXP3+ Treg cells to mediate the immune escape of solid tumours. Several mechanisms of Treg cells have been reported to help tumour to avoid immune destruction. For instance, Treg cells can promote the formation of immune suppressive microenvironment. Treg cells express chemokine receptors (e.g., CCR4, CCR8, CCR5, GPR15) and are recruited to the tumour site by chemokines produced by diverse cells within TME. Treg cells secreted immunosuppressive cytokines, TGF-β, and VEGF, which not only promote the conversion of Tconv cells to Treg cells, but also suppress Teff cells and APCs function. Treg cells constitutively express CTLA-4, while downregulate the expression of CD80/CD86 in APCs (through trans-endocytosis), thereby depriving co-stimulatory signals to responder T cells. Meanwhile, Treg cells inhibit the function of DCs through LAG-3 and MHC II interactions. For metabolic adaptation, Treg cells could converse ATP to adenosine by CD39 and CD73, which directly inhibits A2AR mediated Teff cells function. Cells within TME could also be killed by Treg cells secreted granzyme and perforin. CCL, C-C motif chemokine ligand; CCR, C-C motif chemokine receptor; GPR, G protein-coupled receptor; Tconv, conventional CD4 T cell; TGF, transforming growth factor; IL, interleukin; VEGF, vascular endothelial growth factor; CTLA-4, cytotoxic T-lymphocyte associated protein 4; MHC, major histocompatibility complex; FGL1, fibrinogen like 1; Teff, effector T cell; A2AR, adenosine A2A receptor; LAG-3, lymphocyte activating 3.

FOXP3+ Treg cell-mediated immunosuppression is also executed by the release of multiple immunosuppressive cytokines. IL-10, IL-35, and TGF-β (75, 76) inhibit the function of APCs and Teff cells, while granzymes and perforin directly kill NK and CD8+ T cell (77, 78). Recent studies have also given special attention to T regulatory cells-derived extracellular vesicles and their ability in generating immune tolerance through effector T cells and DCs (79–81). Finally, Treg cells express a higher level of IL-2R α chain (CD25) and can compete with effector T cells for limited IL-2 in TME (82, 83), thereby robbing essential cytokine for the survival of effector T cells. All the above-mentioned studies further provide the mechanistic basis for FOXP3+ Treg cells promoted immune escape of cancer (as shown in **Figure 2**).

### FOXP3+ Treg cells and tumour-promoting inflammation

The inflammatory response could help our body to remove the necrotic tissue and tumours, so it was once considered as a beneficial anti-tumour immune response. However, subsequent studies have demonstrated that the inflammatory process in TME can also lead to the emergence of tumour invasive metastasis, angiogenesis and other tumour-promoting features (84). FOXP3+ Treg cells may play an important role in regulating the balance of tumour inflammation. Traditionally, Treg cells are believed to be the main anti-inflammatory cells in humans, which suppress the function of immune cells and reduce the inflammatory response, resulting in a poorer prognosis in cancer patients. However, more recent studies have revealed the existence of different tissue resident FOXP3+ Treg cell subsets in CRC, in contrast to the classical Treg cell immunosuppressive function, can also exhibit a pro-inflammatory response profile and thus influence the development and progression of CRC (85). Saito and colleagues have grouped CRCs into two types, based on the proportion of FOXP3(lo) non-suppressive T cells (85). FOXP3(lo) Treg cells are distinguished from FOXP3 (+) T cells in the absence of the naïve T cell marker CD45RA, FOXP3 instability, and enhanced secretion of inflammatory cytokines (e.g., IFN-γ) by the FOXP3(lo) Treg subset (85). CRCs patients with abundant FOXP3(lo) Treg cell infiltration are predicted to have better survival. Mechanistically, Fusobacterium nucleatum, and possibly other intestinal bacteria mediate tumor tissues’ production of inflammatory cytokines (e.g., IL-12, TGF-β, and TNF-α) (85–90), thereby affecting the heterogeneity of tumour-infiltrating Treg cells in CRCs and facilitating the expansion of pro-inflammatory FOXP3(lo) non-Treg cells that, in turn, enhances anti-tumour immunity and inhibits tumour formation (85).

In Colitis-Associated Colorectal Cancer (CAC), inflammation is a key driving factor in tumourigenesis and progression. Under extensive pro-inflammatory conditions, FOXP3+ Treg cells may be redirected to a Th17 response by inflammatory cytokine IL-6 together with TGF-β (91). In particular, FOXP3+IL-17A+ T cells accumulate in the colon of patients with ulcerative colitis and CACs. CAC patients with higher FOXP3+ Treg cell levels have a poor prognosis (92). Treg cells co-expressing the transcription factors FOXP3 and IL-17A-related transcription factor RORγt in the dysplastic areas of IBD patients (93). Tumour-infiltrating FOXP3+RORγt+ Treg cells suppress FoxO3 in DCs, leaving IL-6 expression uncontrolled. At the same time, high IL-6 level stimulates STAT3 activation and proliferation of dysplastic cells (93–96). RORγt inhibition in FOXP3+RORγt+ Treg cells suppresses IL-17A production and prevents inflammatory cytokine-induced destabilization of FOXP3 expression induced by pro-inflammatory cytokines (97). Also, inhibition of RORγt increases Th17-like Treg cells’ production of IL-10, thereby enhancing the inhibition of myeloid inflammatory factors (97). Our recent studies in a colitis-associated colorectal mouse model have shown that the inhibition of the MondoA-TXNIP regulatory axis attenuates the immunosuppressive function of Treg cell and induces Treg cells’ expression of Th17 signature genes in a glycolytic metabolic pattern, thus further promoting Th17-type inflammation in the colorectal TME (98). IL-17A expressing Treg cells may cause CD8+ T cell exhaustion by IL-17A, which could accelerate colorectal carcinogenesis and tumour progression. Notably, the use of IL-17A-blocking antibodies could slow the progression of AOM-DSS-induced colorectal cancer and reduce the susceptibility to colorectal cancer in MondoA-deficient mice. Combined treatment with anti-IL-17A and anti-PD-1 antibodies further reduces the size of colorectal tumours in animal model. Interestingly, it has been found that GPR15 expression on T cells also enhanced IL-17 secretion. Gene expression analysis shows that GPR15+ Treg cells have a Th17-like phenotype, leading to the production of IL-17 and TNF-α in AOM/DSS mouse model (60).

Interestingly, during tumour development, CD4+T cells may progressively transdifferentiate into IL-17A+ FOXP3+ and ex-Th17 IL-17A- FOXP3+ T cells (99). FOXP3-expressing subsets possess immunosuppressive function. IL-33, induced in transformed epithelial cells of CRC, is an important trans-differentiation regulator. IL-33/ST2 signaling suppresses IL-17A production and potentially promotes the conversion of IL-17-producing CD4+ T cell types to IL-17-negative (RORγt−) ST2+ FOXP3+ Treg cells, modifying the inflammatory signals within the tumour microenvironment to promote CRC (100).

Taken together, the pro-inflammatory tumour microenvironment, whether intrinsic or induced, may influence the phenotype and function of Treg cells, which consequently, exert anti- or pro-tumourigenic inflammatory responses.

### FOXP3+ Treg cells and the new hallmark of cancer: Polymorphic microbiomes

The “Polymorphic microbiomes” is listed as a new hallmark of cancer (5). Although increasing evidence has shown microbiomes play critical roles in carcinogenesis, and the immune system is closely associated with microbiomes, the relationship among tumour, Treg cells, and microbiome is still largely unclear (101).

The association between Treg cells and microbiomes is mainly explored in colorectal cancer, for large proportion of microorganisms reside in the human gastrointestinal system (102). The immune-suppressive capacity of tumour-infiltrating Treg cells and the M2 subset of tumour-associated macrophages (TAM) are closely correlated with intestinal microbiota in colorectal cancer patients (103). FOXP3+ Treg cells could intervene in the protective process of fecal microbiota transplanted colitis-associated colon cancer mice model (104). GPR109a signaling could also induce the differentiation of IL-10-producing Treg cells (105). The combination of Lactobacillus acidophilus lysate and anti-CTLA-4 therapy could enhance anti-tumour immunity in a mouse model of colon cancer, accompanied with increased CD8 + T cells and effector memory T cells, but decreased Treg cells and M2 macrophages (106).

Apart from CRC, Treg cells and microbiomes also engage in other cancers. High blood butyrate and propionate levels affect anti-CTLA-4 therapy efficacy in mouse model and melanoma patients, along with increased Treg cell proportions, reduced DC and effector T cell activation, and lower responses to IL-2 (107). Probiotics modulate the gut microbiome composition to produce anti-inflammatory metabolites and promote the differentiation of anti-inflammatory IL-10-producing Treg cells, which may help to against hepatocarcinoma (108). Selected Bacteroides spp. (such as B. fragilis, B. thetaiotaomicron) can modulate colonic RORγt+ Treg cells through the bile acid receptor VDR (vitamin D3 receptor), which may be of great significance in treating gastrointestinal and hepatic cancers (109).

FOXP3+ Treg cells may also facilitate carcinogenesis induced by several microbiomes. In gastric, mycobacterial infection could aggravate *Helicobacter pylori*-induced gastric preneoplastic pathology *via* inducing Treg cells (110). Moreover, intratumour microbes are thought to create a tolerogenic program with lower proportions of tumour-infiltrating lymphocytes (TILs) but increased Treg cells in various types of cancers including colorectal, pancreatic, breast, and lung cancers (111–118).

### FOXP3+ Treg cells and the classic hallmarks of cancer: metastasis and invasion

As invasion and metastasis are classical cancer markers, emerging evidence suggests Treg cells also play a role in promoting tumour metastasis *via* multiple manners (5, 119). The levels of FOXP3+ Treg cells are strongly associated with cancer metastasis in various human cancers (120). An Increasing ratio of Treg/Th2 can promote the metastasic progression of hepatocellular carcinoma (121). FOXP3+ Treg cell levels in the peripheral blood of NSCLC patients increase with tumour stage and peak in metastatic patients (117). Increased FOXP3+ Treg cells have also been linked to a higher risk of metastasis in other cancers, including breast, ovarian, prostate, thyroid, gastric, colorectal, and skin cancers (114, 115, 118, 119).

The underlying mechanisms that contribute to the increase of tumour-infiltrating FOXP3+ Treg cells could be categorised into three major pathways. Firstly, organs susceptible to be invaded and metastasized tend to contain more FOXP3+ Treg cells (120). For instance, lung tissue could induce more Treg cells through the upregulation of prolyl-hydroxylase (PHD) proteins and a local reduction of HIF1α, which are correlated with increased intrapulmonary metastasis (122). On the other hand, bone marrow has a relatively hypoxic environment, which contributes to higher Treg cell frequency during bone metastasis of tumours (123). FOXP3+ Treg cells also promote osteogenesis by suppressing osteoclast differentiation and function, a process that may favour the bone metastasis of prostate cancer (124). Secondly, the tumour locus can recruit Treg cells to build an immune-suppressive environment for tumour progression and metastasis. For instance, elevated levels of PGE2 in TME could lead to the recruitment of FOXP3+ Treg cells, which increase the risk of bone metastasis (125). Inhibition of the CXCL12/CXCR4 axis in combination with IDO1 blockage could reduce Treg cell and bone metastasis in breast cancer model (126). After the occurrence of tumour metastasis, breast cancer cells could stimulate lung tissue to secrete CCL17 and CCL22, which attract CCR4-positive Treg cells to accumulate in lung tissue, and thus facilitating lung metastasis of breast cancer (127). Thirdly, Treg cells can promote tumour invasion and metastasis directly. Tan et al. have found Treg cells to express a higher level of RANKL than Tconvs and stimulated pulmonary metastasis of human RANK (+)breast cancer cells, and blocking this pathway can reduce the frequency of pulmonary metastasis (128). Oh et al. have reported, in mouse model, increased invasive and metastatic potential of melanoma owing to the direct contact between melanoma cells and Treg cells. Elevated TGF-β produced by Treg cells induces the epithelial-to-mesenchymal transition (EMT), leading to increased lung metastasis (129).

### FOXP3+ Treg cell function in tumour angiogenesis

Inducing angiogenesis is thought to be one of the mechanism to meet the demand of nutrients and oxygen of cancer and evacuate metabolic wastes and carbon dioxide from TME (5). Recent studies suggest that FOXP3+ Treg cells may also play a functional role in tumour angiogenesis directly or indirectly to promote carcinogenesis (130–133).

FOXP3+ Treg cells can intervene in cancer angiogenesis in two ways: through the VEGF pathways or the modulation of other immune cells with inflammatory cytokine release (134). VEGF family is closely related to blood vessel formation (135). Multiple studies reported the association between FOXP3+ Treg cells and VEGF in cancer patients and *in vivo* tumour animal models (136–142). Hypoxia-induced CCL28 may recruit intratumoural FOXP3+ Treg cells, which can upregulate VEGFA levels to promote angiogenesis directly in ovarian cancer (138) and breast cancer (142).

In addition to VEGF pathways, Treg cells can induce cancer angiogenesis *via* regulating other immune cell functions. For example, Casares et al. reported a reduction of Treg cells levels can induce IFN-γ produced by effector CD4 T cells to decrease tumour angiogenesis and enhance anti-tumour response (143). Beatty et al. also emphasized the critical role of IFN-gamma-dependent inhibition of tumour angiogenesis by tumour-infiltrating CD4+ T cells (144).

On the contrary, cancer angiogenesis could conversely exert an effect on tumour-infiltrating FOXP3+ Treg cells. Numerous clinical studies have demonstrated that antiangiogenic therapy, blocking VEGFR, used in human cancers is associated with a reduction of tumour-infiltrating FOXP3+ Treg cells (145–147). VEGF could promote FOXP3+ Treg cell migration and its immunosuppressive function, but the detailed mechanisms underlying VEGFR blocking therapy and tumour-infiltrating FOXP3+ Treg cells reduction are still unclear (148–150).

### FOXP3+ Treg cells and the newly proposed hallmark of cancer: Cell senescence

Senescent cells are recently proposed as a new and emerging hallmark of cancer (6). Cell senescence is an irreversible cell cycle arrest caused by various factors including: telomere shortening, DNA damage, cellular stress, and oncogenes’ activation (151, 152). In solid tumour tissues, the senescence of immune cells (e.g.,macrophages and effector T cells) is associated with increased tumour malignancy, while the senescence of cancer cells is linked to the suppression of cancer progression (151, 152).

**Figure 3:**
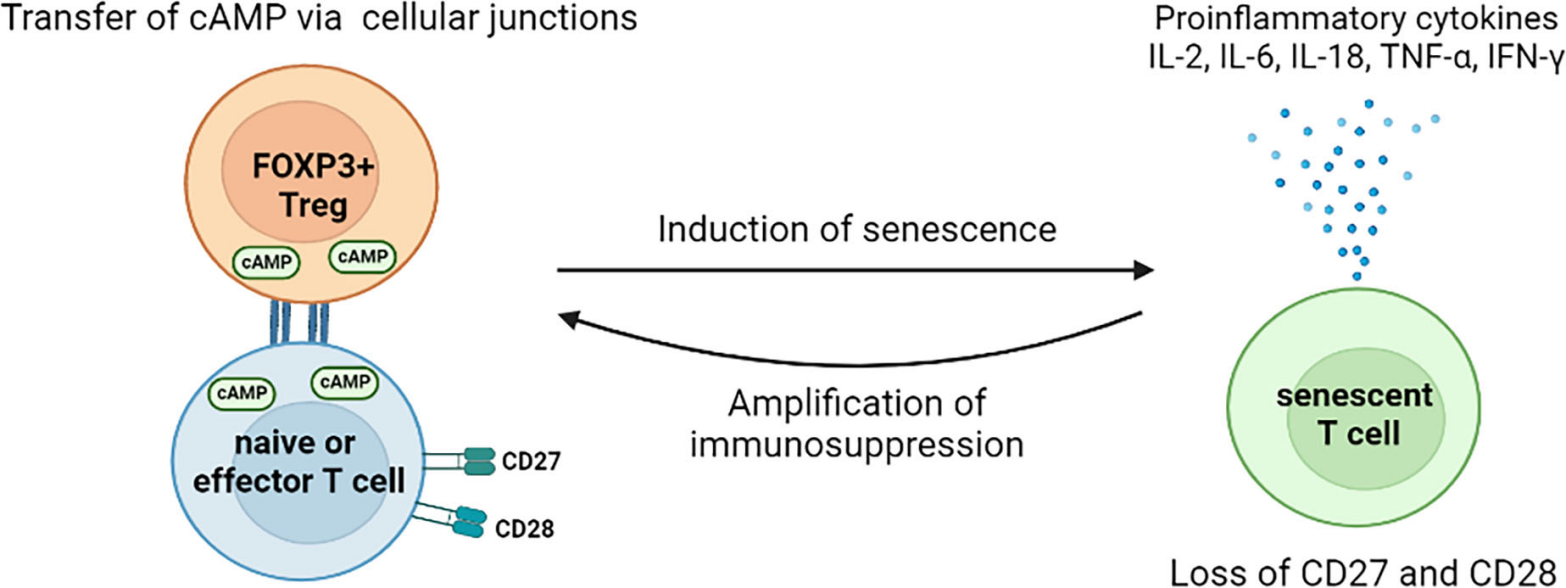
Mechanisms for FOXP3+ Treg cells to induce T cell senescence in the tumour microenvironment. The direct transfer of cAMPs, by Treg cells via cell junctions, induces the senescence of naïve and effector T cells. The induced senescent T cells cease the expression of CD27 and CD28 but increase the secretion of pro-inflammatory cytokines. Thus, those senescent T cells exhibit immunosuppressive features and argument the immunosuppression within TME. So far, no study of direct effect of tumour-associated Treg cells on tumour cell senescence has been found. However, Treg cells might mediate the senescence of tumour cells indirectly. For instance, the Treg cells induced senescent T cells exhibit unique SASP, which is characterized by augmented release of cytokines, chemokines, proteases, and metabolic wastes. The accumulation of these molecules as well as low glucose availability, caused by hyper-glycolysis of Treg cells, create a stress environment, thus may facilitate thesenescence of tumour cells. cAMP, cyclic adenosine monophosphate; IL, interleukin; TNF, tumour necrosis factor; IFN, interferon; TME, tumour microenvironment; SASP, senescence-associated secretory phenotype.

FOXP3+ Treg cells have recently been reported to induce effector T cell senescence by metabolic competition (153). The senescent T cells are characterized by the elevated expression of senescence-associated β-galactosidase (SA-β-gal), decreased expression of CD27 and CD28, and acquired immune suppressive capacities *via* the production of TGF-β and IL-10 (153–157). Tumour-infiltrating FOXP3+ Treg cells exhibit higher glycolysis, which hastens glucose consumption and reduces glucose availability within TME (158). Low concentrations of glucose alone can significantly induce the senescence of both CD4+ and CD8+ T cells (153). The induction of T cell senescence, by FOXP3+ Treg cells mediated glucose insufficient, is initiated *via* the activation of the AMP-activated protein kinase (AMPK) (159). The activated AMPK increases the phosphorylation of p53, facilitates the accumulation of p21^WAF1^, promotes the expression of p27, inhibits the activities of telomerase, and reduces the expression of key components in the Toll-like receptor signalosome (160, 161). In addition, the accumulation of cyclic adenosine monophosphate (cAMP), the metabolic product of Treg cells, is also an important inducer of T cell senescence (159). Treg cells are able to transfer cAMP into T cells *via* tight junctions,and the elevated intracellular level of cAMP in T cells triggers the nuclear kinase ataxia-telangiectasia mutated (ATM) protein associated DNA damage, which ultimately leads to T cell senescence (159, 162). Persistent DNA damage signaling provokes the secretion of senescence-associated inflammatory cytokines, IL-2, IL-6, IL-8, TNF-α, and IFN-γ, which induce more T cell senescence within the suppressive TME (as shown in **Figure 3**) (159). The accumulation of immune suppressive cells enables tumour cells to escape from anti-tumour immune responses (163). However, the effect of Treg cells in inducing the senescence of tumour cells is yet to be illustrated.

### Metabolic connection between FOXP3+ Treg cells and cancer cells in the tumour microenvironment

Although immune receptors, signaling proteins, and transcription factors have participated in T cell responses, cellular metabolism has been recognized as one of the core determining factors for the survival, proliferation, and function of T cells. In general, immunosuppressive FOXP3+ Treg cells are more dependent on β-oxidation than glycolysis, compared with effector T cells (164, 165). However, lactic acid may provide metabolic support to tumour-infiltrating FOXP3+ Treg cells in highly glycolytic TME (166, 167). The ablation of lactate transporter MCT1 in B16 melanoma inoculated Slc16a1f/f Foxp3cre mice leads to decreased tumour growth, indicating the immunosuppressive function of Treg cells may be closely related to their ability of ingesting lactate acid (167). Tumour-infiltrating FOXP3+ Treg cells may convert lactic acid to pyruvate, which is then converted into malic acid and citric acid that ultimately participates in the tricarboxylic acid cycle (167). Further study has shown that a high lactic acid environment allows effector Treg (eTreg) cells to use MCT1 to uptake lactic acid, which upgrades the level of PD-1, leading to the functional and phenotypic changes of eTreg cells (166). In trials comparing the effects of high glycolysis tumours with low glycolysis tumours on CTLA-4 immunotherapy, the therapeutic effect of low glycolysis tumours is found to be more pronounced (168). Our recent studies have also found that the deletion of the MondoA-TXNIP transcriptional regulatory axis allows Treg cells to increase the expression and cell membrane localization of glucose transporter Glut1 for stronger glucose uptake and glycolytic metabolic capacity (98). Inhibiting mitochondria is liable to weaken Treg cells function (169–172). In Treg cells, the loss of mitochondrial transcription factor A (TFAM) is important for mitochondrial respiratory chain activity, impairs the accumulation and cell lineage stability of the tumour-infiltrating Treg cells, and thus, inhibits tumour growth (169, 170). Eliminating Treg cell-specific mitochondrial complex III increases DNA methylation, as well as enhances the accumulation of metabolites 2-hydroxyglutaric acid (2-HG) and succinic acid, thereby inhibiting Treg cells function (172). FOXP3+ Treg cells lacking transcription factor c-Myc have disrupted mitochondrial oxidative metabolic process, which decreases the accumulation and functional activation of Treg cells (171). In addition, there are other pathways related to the metabolic regulation of tissue-resident FOXP3+ Treg cells. For instance, glucose metabolism and glycolysis are selectively inhibited by TLR8 activation in tumour-infiltrating Treg cells (158). Moreover, CD36 expression on tumour-infiltrating Treg cells may mediate the uptake of long-chain fatty acids. Although the knockout of CD36 reduces FOXP3+ Treg cells within tumours, the preservation of peroxisome proliferation activation receptor-β (PPAR-β) signal-dependent mitochondrial adaptability leads to the inhibition of tumour growth (173). Inhibition of fatty acid binding protein 5 (FABP5) leads to changes in mitochondria that enhance the inhibitory capacity of FOXP3+ Treg cells (174). Besides, redox homeostasis is thought to modulate development and function of Treg cells (175, 176). Previous studies have demonstrated that increased Treg cells in tumour sites may be attributed to their increased antioxidative capacity (177, 178). Furthermore, scientists have also paid more attention to the association between Treg cells and redox homeostasis in TME. Thomas-Schoemann et al. have shown arsenic trioxide could increase anti-tumor immune response by decreasing Treg cell numbers. This Treg cell reduction is mediated by oxidative and nitrosative stress (179). Wang et al. have demonstrated that antioxidant protein thioredoxin (TRX) enhances Treg cell infiltration in melanoma, which in turn decreased anti-tumor immune reactions. Recently, Xu et al. have reported that glutathione peroxidase 4 (Gpx4) could prevent Treg cells from lipid peroxidation and ferroptosis in regulating immune homeostasis and anti-tumor immunity (180).

## Conclusion and prospective

FOXP3+ Treg cells in the tumour microenvironment are regulated at multiple levels, which include Treg cell instability (181–183), Treg cell plasticity (184, 185), and tissue Treg cell specificity (186–188). Tissue-resident Treg cells maintain tissue homeostasis and improve tissue repair to prevent inflammation-induced cancer generation. While, within TME, Treg cells repress the anti-tumour immune responses. Treg cells also influence other hallmarks of tumour through cytokines or or other ligands to activate multiple signal pathways, for example, TGF-β is shown to promote tumour metastasis. Tumour cells recruit Treg cells through chemokines, cytokines, and metabolic regulation. Single-cell sequencing and FACS data indicate that in the tumour site there are different Treg cell subsets showing different functions, cytokine expression, and relationships with patient prognosis.

The efficacy of immunotherapy with immune checkpoint antibodies or engineered T cells, especially CAR-T cells, is also regulated by the tumour-infiltrating Treg cells. Several new strategies may be developed in the future to treat tumour by targeting Treg cells. First, develop dual-antibodies to suppress the function of tumour-infiltrating Treg cells; second, generate CAR-T cells resistant to the suppression of Treg cells; and last but not least, convert the suppressive Treg cells into Th1 or Th17-like Treg cells, which may improve their anti-tumour activity. Our understanding of the mutual regulation between tumour-infiltrating FOXP3+ Treg cells and the key hallmarks in solid tumours will provide new clues for generating engineered T cells to cure cancer patients.

## Author contributions

YQ and SK wrote the article. JC, ZQ, WZ and YY designed and drew the figures. DM, GZ, KW, DL and BL proofread this article. All authors contributed to this article and approved the submitted version.

## Funding

Our research is supported by National Key R&D Program of China 2019YFA09006100; National Natural Science Foundation of China grants 32130041, 81830051 and 31961133011; Innovative research team of high-level local universities in Shanghai SHSMU-ZDCX20210601; Key Laboratory of Cell Differentiation and Apoptosis of Chinese Ministry of Education, Shanghai Frontiers Science Center of Cellular Homeostasis and Human Diseases. Shenzhen Science and Technology Program KQTD20210811090115019.

## Conflict of interest

Author BL is a co-founder of Biotheus Inc and chairman of its scientific advisory board.

The remaining authors declare that the research was conducted in the absence of any commercial or financial relationships that could be construed as a potential conflict of interest.

## Publisher’s note

All claims expressed in this article are solely those of the authors and do not necessarily represent those of their affiliated organizations, or those of the publisher, the editors and the reviewers. Any product that may be evaluated in this article, or claim that may be made by its manufacturer, is not guaranteed or endorsed by the publisher.
